# A Portable Waterproof EEG Acquisition Device for Dolphins

**DOI:** 10.3390/s21103336

**Published:** 2021-05-11

**Authors:** Yanchao Yu, Ni Li, Yan Li, Wentao Liu

**Affiliations:** 1 School of Automation Science and Electrical Engineering, Beihang University, Beijing 100191, China; marston2yu@buaa.edu.cn (Y.Y.); lini@buaa.edu.cn (N.L.); 17374039@buaa.edu.cn (W.L.); 2 Beijing Advanced Innovation Center for Biomedical Engineering, School of Biological Science and Medical Engineering, Beihang University, Beijing 100191, China

**Keywords:** dolphin, EEG acquisition device, brain interface

## Abstract

The acquisition and analysis of EEG signals of dolphins, a highly intelligent creature, has always been a focus of the research of bioelectric signals. Prevailing cable-connected devices cannot be adapted to data acquisition very well when dolphins are in motion. Therefore, this study designs a novel, light-weighted, and portable EEG acquisition device aimed at relatively unrestricted EEG acquisition. An embedded main control board and an acquisition board were designed, and all modules are encapsulated in a 162 × 94 × 60 mm^3^ waterproof device box, which can be tied to the dolphin’s body by a silicon belt. The acquisition device uses customized suction cups with embedded electrodes and adopts a Bluetooth module for wireless communication with the ground station. The sampled signals are written to the memory card on board when the Bluetooth communication is blocked. A limited experiment was designed to verify the effectiveness of the device functionality onshore and underwater. However, more rigorous long-term tests on dolphins in various states with our device are expected in future to further prove its capability and study the movement-related artifacts.

## 1. Introduction

Animal electrophysiological signals, including electrocardiogram (ECG), electromyography (EMG), electroencephalogram (EEG), and electrocorticogram (ECoG), have been widely adopted by studies on animal brain functions and medical drug toxicity [1,2]. Former experiments on the electrophysiology of terrestrial mammals, including rats, cats, and monkeys, have proven the feasibility and effectiveness of electrophysiological signal recording [3,4,5]. However, the electrophysiological signal recording for aquatic animals is still troubled by the voltage inference and inadequate contact of the electrode caused by flowing liquid and animal movements, according to studies on fish and aquatic mammals [6,7,8]. 

Among all experimental animals, the dolphin is regarded as an excellent subject in electrophysiology study because of their unique auditory system and well-developed brain, which closely resembles the human brain in both size and functionality [9]. Recent studies have revealed that dolphins possess a highly differentiated central nervous system, for example, the hypertrophic auditory pathway is responsible for the sonar system and the brain stem nuclei for the visual system [10]. The complex brain characteristics suggest that the dolphin is a highly intelligent animal and may present unpredictable and uncontrollable behaviors, which could be an underlying obstruction for electrophysiological signal acquisition. Another factor that may have a huge impact on signal acquisition is that dolphins can swim at a sustained speed of 7.2–9.3 m/s in a natural environment [11]. In such a drastic movement, a more flexible and reliable collecting scheme is needed.

Considering the serviceability and stability of aquatic animal electrophysiological signal recording during movement, an invasive recording procedure is preferred by some researchers. As early as 1977, Mukhametov et al. conducted an invasive experiment to study the ECoG and EMG of dolphins [12]. In the experiment, intracortical electrodes are implanted into the drilled bone aperture under local anesthesia and EMG electrodes are designed as a harpoon shape to be fixed into neck muscles and extraocular muscles. Similar electrode implantation procedures are applied to aquatic mammals, including dolphins, fur seals, and white whales, which prove the technical feasibility of invasive recording [7,8,13]. Despite the invasive method collecting pure signals due to the close distance between the electrodes and the brain, it requires certain surgery techniques, such as animal anesthesia, and suffers greater clinical risks, such as infection and brain damage.

The noninvasive method, in contrast to the invasive one, records signals from electrodes placed on the scalp surface without any surgery and implantation so that consecutive and repetitive experiments are possible. The non-invasive recording system is generally composed of multiple EEG electrodes embedded in suction cups, a signal amplifier, an A/D converter, and a ground station. Lee et al. and Cho et al. designed and developed an EEG recording system dedicated to zebrafish [6,14]. In the recording system, active electrodes and one reference electrode are placed on a flexible printed circuit board (PCB) to better contact with the zebrafish’s round head so that non-invasive recording is possible. Hashio et al. recorded the electroencephalogram related to the different behaviors of a bottlenose dolphin during transportation [15]. An MT-11 telemetry system (NEC Medical System, Tokyo, Japan) and several vinyl chloride suction cups with electrodes placed near the eyeballs, blowhole, and dorsal fin were deployed to record EEG signals from a fixed dolphin and the collected data were digitalized using an RA1300 A/D converter (NEC Sanei, Tokyo, Japan). The experiment, with the following analysis of the power spectrum of the EEG, revealed that the power ratios of the frequency bands are associated with the action of the dolphin. Li et al. constructed a recording system composed of EEG electrodes (Grass Technologies, West Warwick, RI, USA), a Grass CP511 EEG amplifier (Grass Technologies), a DAQmx USB-6251 A/D converter (National Instruments, Austin, TX, USA), and a standard laptop station [16]. In their experiment on the auditory evoked potential (AEP) responses of an Atlantic bottlenose dolphin, the dolphin’s movement range was limited in an 8 × 10 m^2^ floating pen frame with a long cable. 

The shortage of fixing devices means that the animal’s movement is restricted so that animal behavior-related brain studies and consecutive monitoring of animals in a natural environment is scarcely possible. While with the less restricted cable-connected devices, the action of the dolphin is restricted within, emitting sonant and minor head movements only, which is against the natural behavior of the dolphin. The action restriction of such a recording procedure majorly arises from cumbersome onshore devices and cables connecting them with electrodes. If a portable and wearable acquisition device was developed, it could help in collecting more dolphin signals under different behavior patterns, providing more possibilities and a convenient means for humans to understand their intellectual behaviors and feelings. 

Portable EEG recording devices are exemplified by the Neurologger developed by Vyssotski et al., which can record eight-channel, 10-bit EEG signals within an input range of ±750 μV (bandwidth: 1–115 Hz) [17]. Later versions extended the capability to an input range of ±6 mV (Neurologger 2A) and 64 channels and max bandwidth of 100 kHz (Neurologger 3). The device encapsulates recording circuits in a 66 × 36 × 10 mm^3^ PCB while it sustains a typical battery life of 2 days 5 h. The small mechanical dimensions and long battery life make it suitable for portable recording. Though designed for flying pigeon monitoring, recent experiments on walruses and fur seals have proved that with a waterproof casing, the Neurologger can be used for long-term EEG recording for marine mammals [18,19]. In experiments with the Neurologger, invasive electrodes are widely adopted. A mountable non-invasive electrode and a dedicated fixing structure design could be a possible adaptation for dolphin EEG signal acquisition.

Thus, in this paper, we design a novel portable dolphin EEG signal collecting device. The acquisition module is packed into a waterproof box conforming to IP68 standards. Then, the device box is glued with a silicone belt so that the device can be mounted on the back of a dolphin without hindering its movements. To get rid of onshore devices and cables, we separate the whole device into a collecting box and a ground station with Bluetooth communication between them. In case of a communication error, the signals are simultaneously stored into the MicroSD card onboard. With a battery life of up to 8 h and a Bluetooth connection range up to 30 m, the device can be an efficient tool for long-term dolphin EEG acquisition and ultimately support monitoring dolphin behavior in the natural environment.

Our paper is organized as follows: the design of the device is demonstrated in Section 2; the process of our verification experiment is introduced in Section 3; Section 4 presents the experiment signal processing and the result analysis; and we conclude our work in Section 5.

## 2. Design of the Acquisition Device

### 2.1. Device Composition

**Figure 1:**
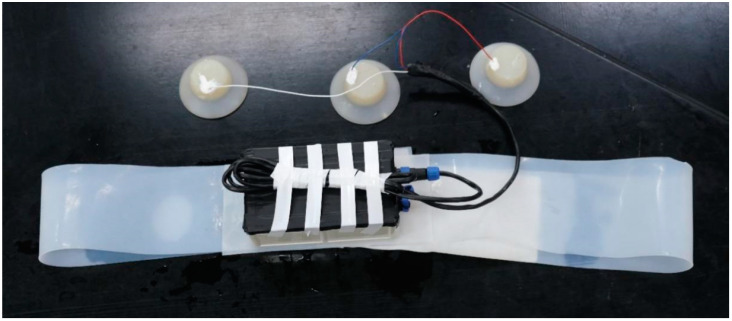
Fully assembled device.

**Figure 2:**
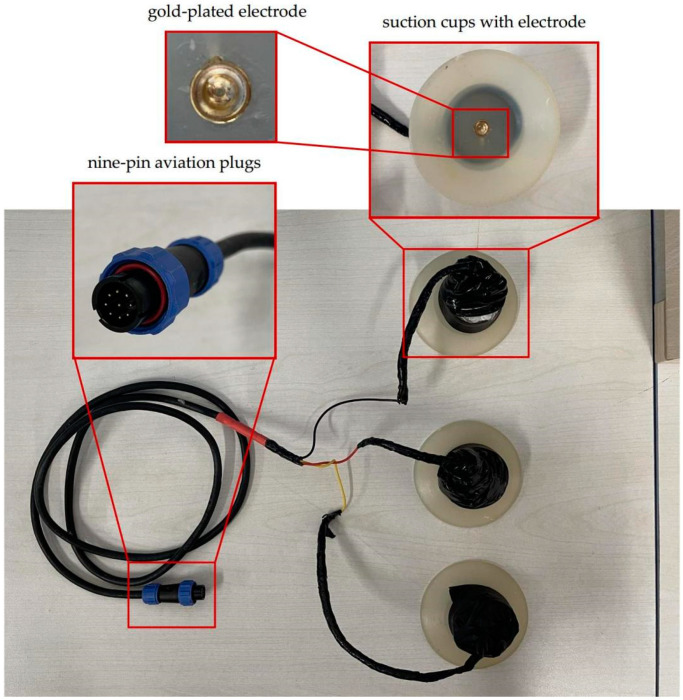
A customized silicone suction cup with an embedded gold-plated EEG electrode.

**Figure 3:**
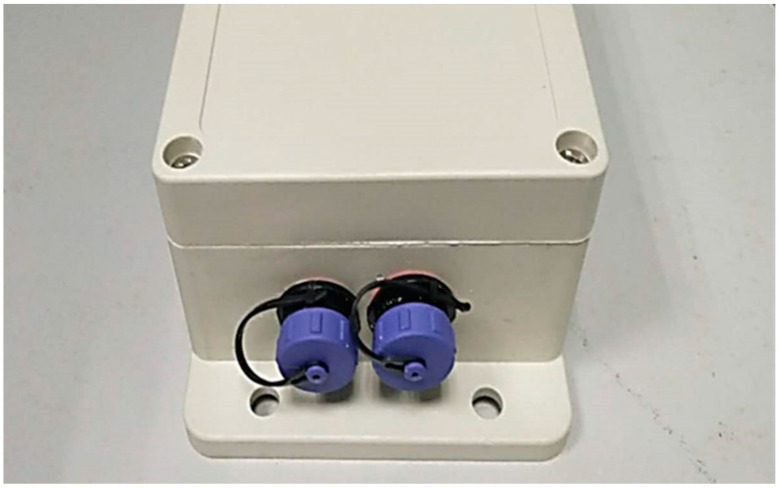
Plug perforated on the side of the device box.

The device is composed of a silicon belt, electrodes, and a device box containing all electrical parts, as shown in Figure 1. Customized silicone suction cups with a diameter of 8 cm were used in our device, in which flat gold-plated EEG electrodes were embedded, as shown in Figure 2. The suction cups are used to stick the electrodes to the skin of dolphins as well as separate the electrodes with outer water environments to avoid current leakage. The electrodes are connected to the device box by wires wrapped with magnetic protective shielding material. The weight of the device box (waterproof rating of IP68) is 247 g, and the external dimension is 162 × 94 × 60 mm^3^. Two nine-pin aviation plugs (Weipu SP-13 with IP68 waterproof rating) are perforated on the side of the device box: the plug on the right is for collecting signals while the one on the left is for charging and copying data, as shown in Figure 3. The belt is made of a silicone rubber sheet, with a width of 100 mm, a length of 1500 mm (adjustable according to the chest circumference of the dolphin), and a thickness of 3 mm.

### 2.2. Electrical Components

**Figure 4:**
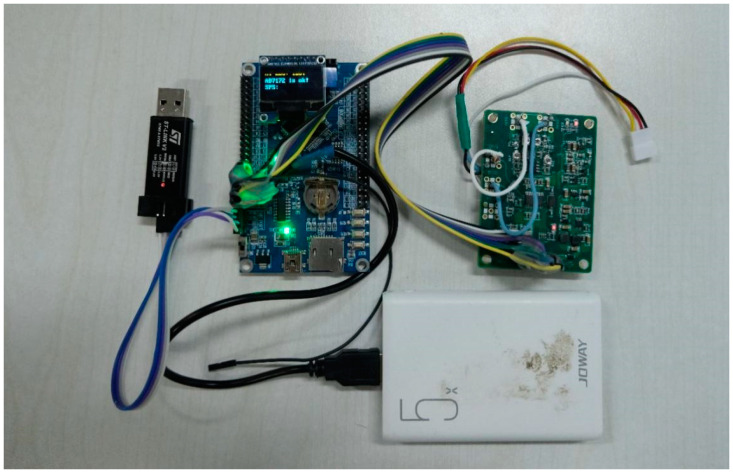
The connection between the electrical parts of our device.

The electrical components include a main control board, an acquisition board, a Bluetooth module, and a 5000 mAh mobile power supply. Their connection relationships are shown in Figure 4. The working time could reach more than 8 h.

**Figure 5:**
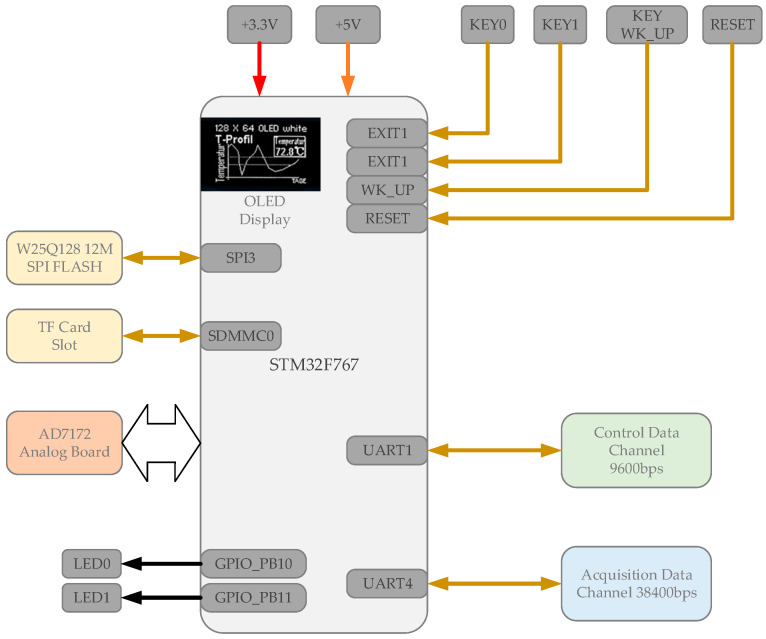
The functional block diagram of the main control board.

The main control board was composed of an stm32f767ZI chip, an ssd1306 display module, a Bluetooth communication module, and its peripheral circuits. Its function includes controlling the collection board, distributing clock frequency, performing Bluetooth communication, circuit self-testing, surface contact resistance measuring, and displaying relevant information in the display module, as shown in Figure 5.

The main control board was responsible for saving data to the flash memory or MicroSD card on the board. It could also transmit data to the ground station synchronously via Bluetooth and display the real-time waveform on the computer.

**Figure 6:**
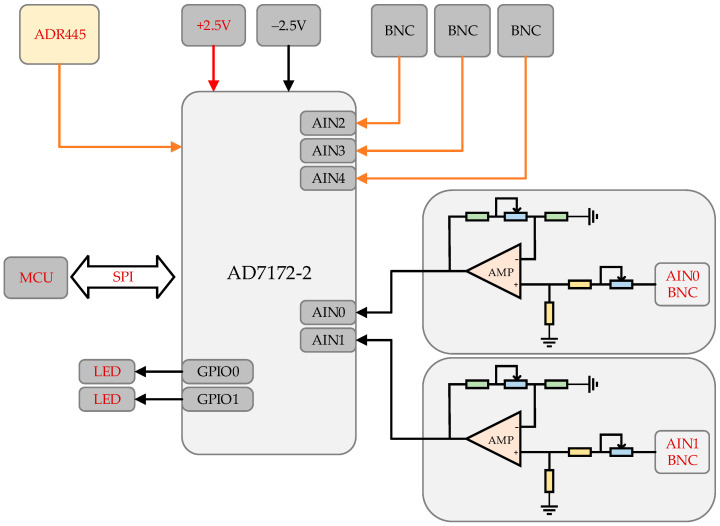
The functional block diagram of the collection board.

The acquisition board consists of an analog-to-digital converter (AD7172-2) and its peripheral circuits. The channels labeled as AIN0-AIN4 have a precision of 24 bits, input range of ±4.5 V, maximum sampling rate of 31 kHz, and a gain factor of 200, with AIN0-AIN1 as EMG channels and AIN2-AIN4 as EEG channels, as shown in Figure 6.

### 2.3. Working Principle

#### 2.3.1. Measurement of the Surface Contact Resistance

**Figure 7:**
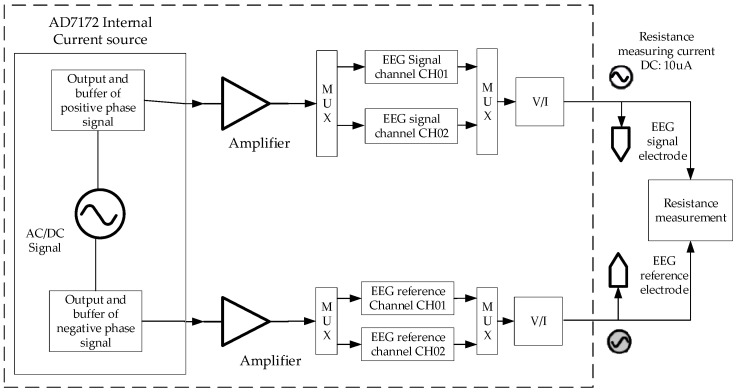
The functional diagram of measuring resistance.

The conductivity between electrodes was checked by measuring the skin surface resistance between two electrodes, which is a prerequisite for later signal acquisition. During the measurement, the internal AD 7172 current sources are connected to the measurement circuit by an analog switch. It provides a current of 10 μA for about 5 ms until accurate resistance can be obtained. The measurement circuit diagram is shown in Figure 7. In our experiment, the surface resistance between the two electrodes was generally about 22–24 kΩ.

#### 2.3.2. Signal Acquisition and Storage

The sampling frequency can reach as high as 31 kHz. The data accuracy will deteriorate when the sampling frequency is too high and a frequency of 500 Hz, 1 kHz, 2.5 kHz, or 5 kHz is recommended. The main control board has a 128 M flash and a MicroSD card slot, which can accommodate an extra memory card with a large capacity.

For the actual needs of this experiment, only one channel is used, although five channels were available. If more than five channels are needed, a second acquisition board can be extended.

We used HC-05 for Bluetooth communication and the baud rate was set to be 38,400 when the sampling rate is 500 Hz. The data is packed and transmitted in frames simultaneously during signal acquisition. For the transmission verification, each frame contained 8 bytes, including two start bytes, one check byte, one frame end byte, and four data bytes. Considering the real-time communication of Bluetooth could be interrupted when dolphins dive into the water, the data would also be written into the memory card. After emerging from the water, the data could be transmitted to the computer via Bluetooth with read-write commands.

### 2.4. Waterproof Measures for Electrical Parts

Since the device works in an environment with water, it is inevitable to consider waterproofing issues. The device box we used is an IP68 waterproof and dust-proof box, with a sealing strip on the inner side of the lid. The cables are connected to the plugs, which are installed on the drilling holes on the device box. The junctions between the device box and the plugs are designed to avoid water seepage. Although the aviation plugs with good water tightness are adopted, leakage could happen if they are not installed tightly. The sizes of the drilling holes should be controlled. The gap between the plug and the hole is sealed with hot-melt adhesive for waterproof capability. Besides, the shielded cables are used to reduce electrical inference.

## 3. Materials and Methods

In order to verify the EEG acquisition capability of the device designed in this paper, we designed a dolphin EEG signal acquisition experiment under different dolphin behavior conditions. The experiment aims to prove the effectiveness of our device by comparing the spectral characteristics of the collected signal with the former research presented in Hashio et al. [15].

The two dolphins used in the experiment belong to Tianzhushan Ocean Park. The animal profiles are as follows: female dolphin numbered BL-8, body length 2.8 m, weight 225 kg; female dolphin numbered BL-3, body length 3.1 m, weight 230 kg. BL-8 was trained for the underwater experiment while EEG signals of BL-3 were recorded onshore. The training times of the two dolphins were 10:00 a.m., 2:00 p.m., and 4:00 p.m., respectively. The experiment was conducted in two circular training pools separated by fences, with a depth of 5 m and a diameter of 6 m. During the experiment, the subject was lured to a training pool or the shore, being isolated to avoid the interference of other dolphins. The experimental process was carried out in strict accordance with the “Guidelines for the Ethical Review of Laboratory Animal Welfare” and the animal experiment regulations of Tianzhushan Ocean Park, under the guidance of trainers.

During the experiment of collecting EEG underwater, the acquisition box was carried by the dolphin with the fastened belt, and three electrodes were tightly attached to the dolphin’s skin with the suction cups. 

The installation process of the acquisition device was as follows:

(1) Install a short silicone plate on the back of the acquisition box with screws into the mounting holes.
(2) Stick the short silicone plate onto the long silicone belt with silicone adhesive (Jl-401ab) and wait for half an hour until completely dry.
(3) Place the circuit board carefully in the device box and tighten the screws of the device box cover, and at the same time connect the experimental electrodes through the aviation plug.
(4) Stick the surface of the transparent cover with electrical tapes to avoid visual interference to the animals, which is brought by the flashing power light on the circuit board.
(5) Adjust the silicone belt length with the help of Velcro on both ends to fit around the dolphin’s chest circumference and place it in front of the dorsal fin to ensure it does not fall off.

**Figure 8:**
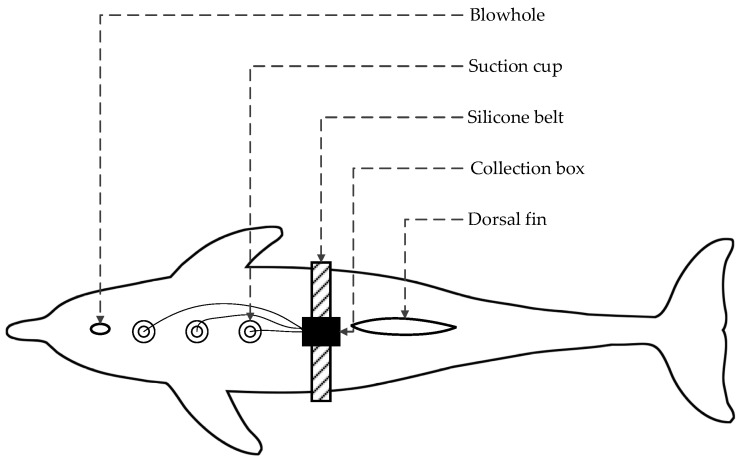
Fixation scheme of the acquisition device.

The positions of the EEG electrodes were determined by the principle of close to the brain and away from the muscles near the mandible and the pectoral fin to avoid EMG influence [15]. Accordingly, the three electrodes were placed in a straight line as follows: the geoelectric electrode G was placed close to the dorsal fin, the positive electrode S was placed close to the blowhole, and the negative electrode M was placed in the middle of G and S, about 15 cm away from the geoelectric pole G. The fixation scheme of the device is shown in Figure 8.

The electrodes were wet with seawater before being placed on skin. Before the experiment, the surface contact resistance of the dolphins was measured. In our experiment, the resistance was about 20 kΩ. Considering the resistance requirement for human brain electrical experiments was below 10 kΩ [20], in the same order of magnitude as that of the dolphin skin, it can be judged that the wetted skin has good conductivity and the biological signals can be collected well without conductive paste.

The device configuration was as follows: the sampling rate was selected as 500 Hz. In the underwater acquisition experiment, the subject dolphin carried out a series of movements under the guidance of the trainer. During the dolphin’s action, the acquisition of EEG signals and a video recording were performed simultaneously. After the acquisition, the EEG signals were marked according to the specific action in the video.

Considering that the real-time signal transmission may be blocked when being underwater, the delayed collected signals can be transmitted to the workstation on the shore after the completion of the experiment and retrieval of the device and belt.

## 4. Signal Processing and Analysis

### 4.1. Collected Signals

**Figure 9:**
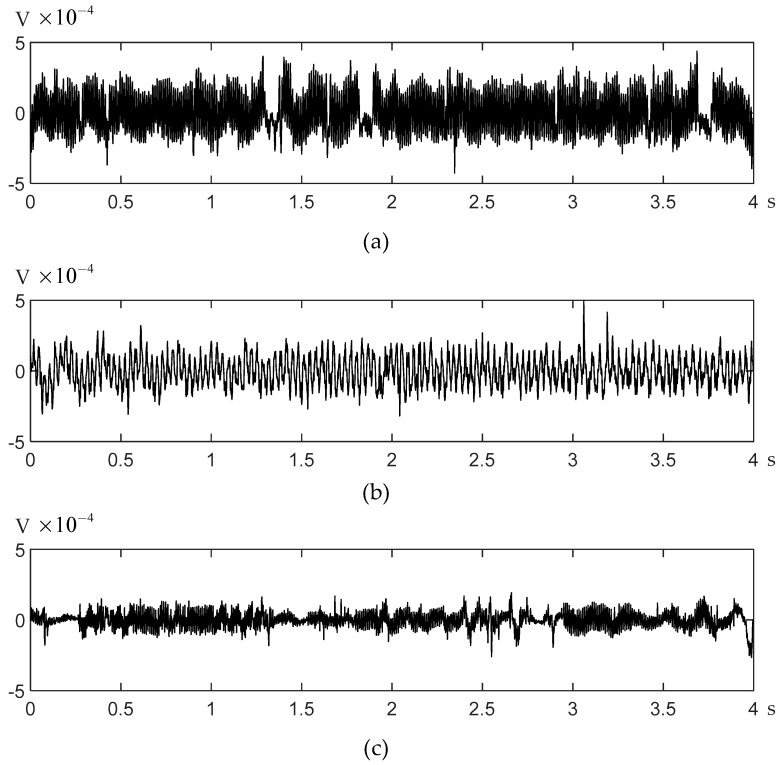
Waveforms of three segments of dolphin EEG signals with label (**a**) UW1, (**b**) UW2, and (**c**) AS.

Three segments of EEG signals were collected in the experiment, and their waveforms are shown in Figure 9. The specific information of the EEG signal is shown in Table 1, including two segments of EEG signals collected underwater, labeled as UW1 and UW2, and one segment of the signal onshore, labeled as AS. 

### 4.2. ECG Artifacts

**Figure 10:**
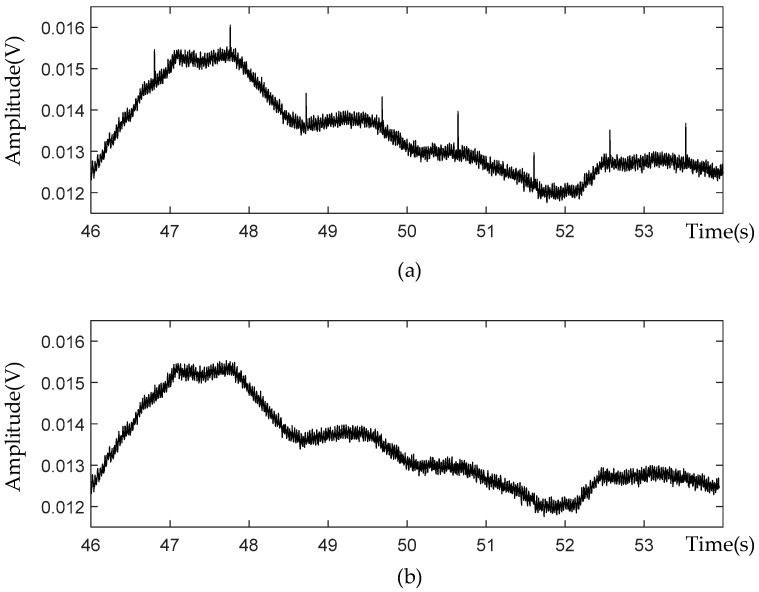
(**a**) EEG signals with ECG artifact, (**b**) EEG signals removing ECG interference.

According to Hashio et al., there may be ECG artifacts in the collected dolphin EEG signals, which are manifested as pulse signals with an interval of about 1.4 s [15]. These artifacts have no obvious frequency characteristics and cannot be removed by a bandpass filter. Therefore, we developed an algorithm to identify the ECG signals based on the observation that the ECG signal appears in a form of two or three consecutive signals and the average amplitude difference of ECG signals with its two neighboring signals is multiple times greater than the mean amplitude difference of the whole signal in a consecutive timeline. Then, the ECG is removed from the signals and its neighboring sequence are jointed. The EEG signal waveforms before and after eliminating the interference of the ECG signal are shown in Figure 10.

### 4.3. Signal Labeling

Before signal analysis, it was necessary to associate the signal with the motion states of the dolphins. We used a video record to aid the association. First, the interested dolphin movements were selected, and each frame of the video was labeled according to its corresponding movement. Then, the video and collected signals were aligned and segmented so that signals were labeled as well. 

It must be mentioned that due to the dolphin’s movements being complicated, we only chose recognizable movements, so the resulting signals are shorter than the original signals after eliminating uninterested motion states. Three motion state-related signals were extracted, including “motionless”- and “swimming”-related EEG from underwater signals and “ashore” EEG from ashore signals. The detailed information of the extracted signals is shown in Table 2. 

### 4.4. Power Spectrum Analysis

Hashio et al.’s research showed that recorded EEG could contain EMG with a frequency band of 60–120 Hz and artifacts coming from breathing and head movement with a frequency below 5 Hz [15]. Therefore, we adopted an FIR bandpass filter with cutoff frequencies of 5 Hz and 40 Hz to filter the EEG signal. 

To reduce randomness and the influence of signal noises, an averaging approach was applied to the power spectrum of signal segments with a 10-s time interval. The EEG signal segmentation is shown in Table 3. 

**Figure 11:**
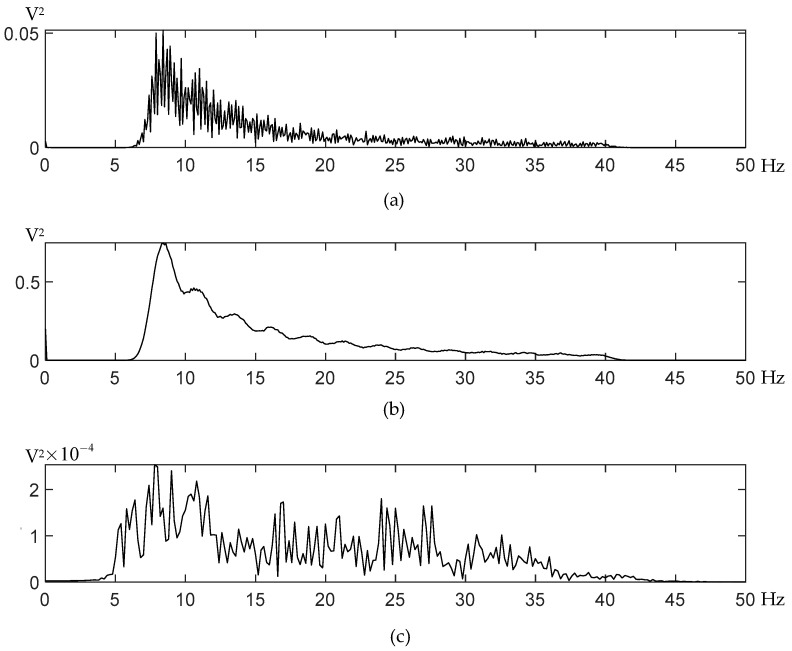
The averaged amplitude-frequency responses of EEG signals recorded from dolphin BL-8 when motionless (**a**) and during swimming (**b**) and dolphin BL-3 recorded ashore (**c**).

The average power spectrums of the EEG signals reveal that the power spectrum distribution of different motion states is distinct with respect to amplitudes and peaks, as shown in Figure 11. After comparing the three underwater EEG power spectrums, it could be concluded that the EEG signal of the dolphin has the following characteristics: (1) The envelopes of the two underwater EEG power spectrums are roughly the same in that the peak of the signal appearing in frequency around 8.4 Hz, and the signal strength decreases as the frequency increases while the difference is that the swimming signal has a greater amplitude. (2) When the dolphin was ashore, the EEG signal of the dolphins was relatively weak compared to the underwater signals. One possible explanation for the amplitude difference between behavior-related signals is movement-related artifacts, which remains to be examined through further inferential testing. This observation (1) is similar to the result presented in Hashio et al., in which the signals of the dolphin when moving its head are significantly greater than the signal in a quiet state [15]. The similarity, to some extent, proved that the signals collected by our device are effective and has potential to be used for further research on dolphin brains.

## 5. Discussion and Conclusions

This paper presents a portable dolphin EEG acquisition device. The Bluetooth technique helps to avoid the obstruction of cables and the miniaturization of the device supports long-term portability, which provides the possibility of collecting dolphin EEG signals in a natural environment. Additionally, we performed a device test experiment and successfully collected the dolphin’s signals in different motion states, with the device and the electrodes staying stable during the experiment.

For the device proposed in this paper, there is still some work that remains to be done.

(1) Perform an additional device test.

In the verification experiment, the dolphin behavior is coarsely classified and during the experiment, the movement of the dolphin is not meticulously designed. Thus, the result drawn from the experiment is limited and inconclusive. An additional long-term device test has to be performed on dolphins with various motion status to verify the capability of the proposed device and examine whether the device is vulnerable to movement-related artifacts.

(2) Improve the performance and control device size.

Compared to the Neurologger proposed by Vyssotski et al., our device still requires improvements in the sampling rate, number of channels, and battery life [17]. In order to achieve a higher precision and input range, as well as simultaneous data communication, a powerful chip and a high-capacity battery was selected, which makes the device. The balance between power consumption and device performance could be improved. The components could be replaced with low-power dissipation chips, and a PCB with a higher integration level would be expected to reduce the device size and power consumption.

(3) Improve the waterproof capability and wear comfort.

The environment of our device test experiment environment was in the pool or on the shore for a short time, which means its deep-water-acquisition functionality was not examined. Under the deep-water environment, it is necessary to improve the waterproof quality, such as using a stainless-steel plug. Moreover, in long-term experiments, a streamlined device box or soft printed PCB that can be directly pasted on the skin would help reduce the resistance during swimming and improve wear comfort.

(4) Improve software and video synchronization.

The bundled ground station software for our device is still a demo version, providing only a basic function, including device self-check, surface skin resistance measurement, start/stop data transmission, erasure of the memory card, and other commands. A further version of the software should be designed to record the experimental time, location, animal, and experimenter profiles. Moreover, our experiment classifies signals by matching video and signals manually, which means an integrated function for synchronizing video and data acquisition should be helpful for future work.

## Figures and Tables

**Table 1:** The sampled EEG of the dolphins.

Label	Signal Length(s)	Scene
UW1	100	Underwater
UW2	90	Underwater
AS	30	Ashore

**Table 2:** Preprocessed EEG signals of various states.

Number	Motion States	Time Length(s)
UW1	Motionless	13.55
UW2	Swimming	54.52
AS	Ashore	30.00

**Table 3:** Summary of the EEG signal segmentations.

Motion States	Number of Segments
Motionless	2
Swimming	6
Ashore	3
